# Role of Lactic Acid Bacteria Phospho-β-Glucosidases during the Fermentation of Cereal by-Products

**DOI:** 10.3390/foods10010097

**Published:** 2021-01-05

**Authors:** Marta Acin-Albiac, Pasquale Filannino, Kashika Arora, Alessio Da Ros, Marco Gobbetti, Raffaella Di Cagno

**Affiliations:** 1 Faculty of Science and Technology, Libera Universitá di Bolzano, 39100 Bolzano, Italy; marta.AcinAlbiac@natec.unibz.it (M.A.-A.); kashika.arora@natec.unibz.it (K.A.); alessio.daros@natec.unibz.it (A.D.R.); marco.gobbetti@unibz.it (M.G.); 2 Department of Soil, Plant and Food Science, University of Bari Aldo Moro, 70126 Bari, Italy; pasquale.filannino1@uniba.it

**Keywords:** phospho-beta-glucosidases, cereal by-products, brewers’ spent grain, lactic acid bacteria metabolism

## Abstract

Bioprocessing using lactic acid bacteria (LAB) is a powerful means to exploit plant-derived by-products as a food ingredient. LAB have the capability to metabolize a large variety of carbohydrates, but such metabolism only relies on few metabolic routes, conferring on them a high fermentation potential. One example of these pathways is that involving phospho-β-glucosidase genes, which are present in high redundancy within LAB genomes. This enzymatic activity undertakes an ambivalent role during fermentation of plant-based foods related to the release of a wide range of phenolic compounds, from their β-D-glycosylated precursors and the degradation of β-glucopyranosyl derived carbohydrates. We proposed a novel phenomic approach to characterize the metabolism drift of *Lactiplantibacillus plantarum* and *Leuconostoc pseudomesenteroides* caused by a lignocellulosic by-product, such as the brewers’ spent grain (BSG), in contrast to Rich De Man, Rogosa and Sharpe (MRS) broth. We observed an increased metabolic activity for gentiobiose, cellobiose and β-glucoside conjugates of phenolic compounds during BSG fermentation. Gene expression analysis confirmed the importance of cellobiose metabolism while a release of lignin-derived aglycones was found during BSG fermentation. We provided a comprehensive view of the important role exerted by LAB 6-phospho-β-glucosidases as well the major metabolic routes undertaken during plant-based fermentations. Further challenges will consider a controlled characterization of *pbg* gene expression correlated to the metabolism of β-glucosides with different aglycone moieties.

## 1. Introduction

Being widespread in various plant and animal ecological niches, lactic acid bacteria (LAB) metabolism is inevitably linked to human nutrition and food bioprocessing, due to their positive impact on sensory and dietary properties of fermented foods [1]. Plant matrices encompass a wide range of health-promoting substrates (e.g., antioxidants, phenolics, fatty acids and dietary fibers). Bioprocessing of a wide range of plant-derived by-products containing a plethora of nutrients is being revealed as a powerful way to exploit and reintroduce vegetable and cereal by-products into the food chain by enhancing their technological and nutritional properties [2].

LAB have the capability to metabolize a large variety of carbohydrates, which in turn enabled their colonization to numerous plant ecosystems and favored the acquisition of genes through horizontal gene transfer (HGT), encoding for specific carbohydrate metabolism and transport [3]. Nonetheless, the efficacy of overall carbohydrate metabolism by LAB relies only on few metabolic routes, strengthening them with an enormous fermentation potential [4]. β-D-glucosidase activity represents an example, which undertakes an ambivalent role during fermentation of plant-based foods related to the release of a wide range of plant secondary metabolites, such as phenolic compounds, from their β-D-glycosylated precursors and the degradation of β-glucopyranosyl-derived carbohydrates [5]. In addition, β-D-glucosidase activity has been positively associated with the flavor of fermented foods by removing undesired bitter compounds and further release of aroma related compounds [6,7]. Encoding genes for this enzymatic activity are widespread among LAB and, more particularly, phospho-β-glucosidases related genes are found in high redundancy within their genomes [8]. Phospho-β-glucosidases catalyze the degradation of phosphorylated glucosides and fibre-related disaccharides, such as cellobiose and gentiobiose, which are activated through the phosphoenolpyruvate (PEP)-dependent carbohydrate phosphotransferase systems (PEP-PTS). This system is more commonly found in obligatory and facultative anaerobes and requires specific cellobiose or generic β-glucosides PTS, which seems to be the substrate-specific part of the catabolic cascade [5,9]. The unexplored potentiality of phospho-β-glucosidase activities and overall carbohydrate metabolism should deserve more attention during the selection of starters for the valorisation of cereal by-products.

Brewers’ spent grain (BSG), the most abundant by-product generated in the beer-brewing process, represents an example of valuable raw material and source of health promoting compounds [10,11]. BSG is a current challenge within cereal by-products valorisation due to the complexity of its indigestible polymers (e.g., hemicellulose fraction) and anti-nutritional factors (e.g., polyphenols) [12].

In this study, we proposed a phenomic screening on the carbohydrate metabolism fluctuations of *Lactiplantibacillus plantarum* and *Leuconostoc pseudomesenteroides* when exposed to a high phenolic and rich-fibre plant matrix, using BSG as model system. The capability of *L. plantarum* and *Leuconostoc* species to grow on similar substrates was previously reported [2,12,13]. To the best of our knowledge, only few studies investigated in depth the phospho-β-glucosidase activity within the overall metabolic strategy undertaken by lactic acid bacteria during fermentation of lignocellulosic substrates [14]. Consequently, this study aims to provide an in vivo phenome assessment of how strains of *L. plantarum* and *Leuc. pseudomesenteroides* shape their metabolism under a BSG ecosystem. Phenotype switching was further complemented and validated through differential gene expression involved in the adopted metabolic strategies mainly related to phospho-β-activities.

## 2. Materials and Methods

### 2.1. Microbial and Biochemical Characterization of Raw-BSG

The company Senson-Viking Malt (Helsinki, Finland) kindly supplied Finnish (FL) brewers’ spent grain (BSG). Chemical composition of the dried FL-BSG as provided by the manufacturer was as follows: 19.8% proteins, 2.9% ash, 55.3% dietary fibers and 9.4% carbohydrates. Dry matter content of the BSG was 25.32% (American Associations of Cereal Chemists approved method 44-15.02). Ten grams of FL-BSG sample was suspended in 90 mL of sterile sodium chloride (0.9%, *w*/*v*) solution and homogenized in a Bag Mixer 400P (Interscience, St Nom, France) at room temperature. Presumptive lactic acid bacteria were determined on Rich De Man, Rogosa and Sharpe medium (MRS, Oxoid, Dublin, Ireland) supplemented with cycloheximide (0.1 g/L), at 30 °C for 48 h under anaerobiosis. Total Enterobacteriaceae were enumerated on Violet Red Bile Dextrose Agar (VRBDA, Oxoid, Dublin, Ireland) at 37 °C for 24 h, total mesophilic bacteria on Plate Count Agar (PCA, Oxoid, Dublin, Ireland) at 30 °C for 48 h, and moulds on Potato Dextrose Agar (PDA, Oxoid, Dublin, Ireland) at 32–35 °C for 48 h.

Yeast cell number was estimated on Malt Extract Agar (MEA, Oxoid, Dublin, Ireland), supplemented with chloramphenicol (0.1 g/L) at 30 °C for 48 h, acetic acid bacteria on Acetobacter Agar (Himedia, Bengaluru, India) supplemented with mannitol at 37 °C for 24 h. *Staphylococcus* and Micrococcaceae on Mannitol Salt Agar (MSA, Biolife, Milan, Italia) at 37 °C for 24 h. Coliforms on Lauryl Sulfate Broth (Sigma-Aldrich, Steinheim, Germany) at 35 °C for 18 h, *Pseudomonas* and *Aeromonas* on GSP Agar (Sigma-Aldrich, Steinheim, Germany), supplemented with Penicillin G (60 mg/L) at 28 °C for 24 h, and enterococci on *Enterococcus* Differential Agar Base (TITG Agar Base, Himedia, Bengaluru, India), supplemented with TTC solution 1% (FD057, Himedia, Bengaluru, India) at 37 °C for 18 h. Total anaerobes and facultative anaerobes on Thioglycollate Agar (TG Agar, Biolife) at 30 °C for 72 h under anaerobiosis and on Tryptic Soy Broth (Biolife) at 30 °C for 24 h under anaerobiosis, respectively. The results were expressed as colony forming units per one gram of BSG (CFU/mL).

Mycotoxins (aflatoxins, fumonisin and zearalenone) were analyzed via TLC (thin layer chromatography) as described in the Official Methods of Analysis (AOAC). Fumonisin B1 was assessed through high-performance liquid chromatography–mass spectrometry (HPLC-MS). The results were expressed as micrograms of mycotoxin per one kilogram of BSG.

The mineral content of FL-BSG (calcium, iron, magnesium, manganese, potassium, and sodium) was analyzed through ICP-MS (inductively coupled plasma mass spectrometry) with MI 2385 rev February 2018 method. The results were expressed as milligrams of minerals per one hundred grams of BSG.

### 2.2. Preparation of Finnish Brewers’ Spent Grain (FL-BSG)-Based Medium

FL-BSG medium was chosen as model system representative of BSG ecosystem. FL-BSG was grinned by a laboratory mill Ika-Werke M20 (GMBH, and Co. KG, Staufen, Germany). Briefly, FL-BSG medium was obtained through a multi-step sequential process. One-hundred grams of FL-BSG were homogenized with 40% of distilled water, incubated for 18 h at 25 °C under stirring conditions (100 rpm), centrifuged (10,000× *g* for 20 min at 4 °C), sterilized by filtration on 0.22 μm membrane filters (Millipore, MO, USA), and stored at −20 °C before use. MRS medium (Oxoid, Dublin, Ireland) was used as the control. The main chemical composition of FL-BSG medium is shown in Table S1 in the Supplementary Material.

### 2.3. Microorganisms and Growth Condition

*Lactiplantibacillus plantarum* PU1 obtained from the Culture Collection of the Department of Soil, Plant and Food Science of the University of Bari Aldo Moro (Bari, Italy) and *Leuconostoc pseudomesenteroides* DSM 20193 obtained from the Leibniz Institute DSMZ (Braunschweig, Germany) were used in this study. These strains were previously characterized for pro-technology (e.g., acidifying and growth capacity) and functional features (e.g., *L. plantarum* PU1 for the ability to increase the antioxidant activity during BSG fermentation and *Leuc. pseudomesenteroides* DSM 20193 to synthetize dextran in different food substrates) [2,15]. The aptitude of *L. plantarum* PU1and *Leuc. pseudomesenteroides* DSM 20193 to ferment BSG was also verified preliminarily [2,12]. Cultures were maintained as stocks in 15% (*v*/*v*) glycerol at −80 °C. Culture inoculum was prepared by harvesting cells during the late exponential growth phase (ca. 8 h) in MRS broth. Cells were washed twice in 50 mM sterile potassium phosphate buffer (pH 7.0). The cell number of *L. plantarum* PU1 and *Leuc. pseudomesenteroides* DSM 20193 used to inoculate FL-BSG medium was ca. 7.0 Log colony-forming units (CFU)/mL. Both strains were cultivated in MRS as a control condition. Incubation was performed at 30 °C for 24 h. Biologically independent triplicates were performed for each condition. Kinetics of growth were determined by measuring the optical density at 620 nm (OD620) (UV1800 Spectophotometer, Shimazu, Japan) and through routine plate count. Plate count data were modelled according to the logistic equation available in grofit R package [16]. A is the maximum cell density reached by the culture at the stationary phase of growth (expressed as CFU/mL), μmax is the maximum growth rate (CFU/mL·h) and λ is the length of the latency phase (expressed in h). The pH was measured by a Crison pH-meter (model 507; Crison).

### 2.4. Phenotypic Microarray Analysis

The phenotypic fingerprints of cells grown on FL-BSG medium and MRS were recorded using the OmniLog Phenotype MicroArray (PM) platform (Biolog, Hayward, CA, USA). Each PM assay was performed on two biological replicates, in accordance with the manufacturer’s instructions. Briefly, cells at the late exponential (LE) growth (after ca. 14 h on FL-BSG medium and ca. 8 h on MRS) were collected, washed in sterile potassium phosphate buffer (50 mM, pH 7.0), and inoculated in PM1 and PM2 microplates, which account for 190 different carbon sources. Kinetic data from PM panels were automatically recorded by the OmniLog reader (Biolog) during incubation at 33 °C for 48 h. Generated longitudinal data were analyzed using the Micro4Food PM pipeline [17]. Briefly, blank subtraction was performed, and metabolic profiles were categorized as active and non-active. Metabolic signals were normalized per replicate and array [18]. After removal of common non-active profiles, metabolic parameters were computed using a free splines method and confidence intervals (CI) were determined through bootstrapping [16].

### 2.5. Determination of Organic Acids and Sugars

Organic acids and sugars were determined every four hours throughout the growth kinetics with a Dionex UltiMate 3000 (Thermo Fisher, Waltham, MA, USA) HPLC apparatus. Organic acids were analyzed by using an Aminex HPX-87H column (Biorad, Hercules, CA, USA) according to the method described by Zeppa et al. [19]. Sugars were determined by using a Spherisorb column (Waters, Millford, MA, USA) as described by Rizzello et al. [20].

The results were expressed as means of three biological replicates analyzed in triplicate ± standard deviations.

### 2.6. High-Performance Liquid Chromatography–Tandem Mass Spectrometry (HPLC-MS/MS) Analysis of Phenolic Compounds

Cell suspensions of *L. plantarum* PU1 and *Leuc. pseudomesenteroides* DSM 20193 grown on FL-BSG media at 30 °C for 24 h were harvested (after different periods of: 0, 2, 4, 6, 8, 12, 16, 20, and 24 h), centrifuged (10,000 rpm for 5 min), and the supernatant was filtered and stored at −20 °C until further use. Separation and identification of phenolics was performed through liquid chromatography–tandem mass spectrometry (LC-MS/MS) analysis according to the method set up and validated by Tlais et al. [21] An ultra-high performance liquid chromatography (UHPLC) Dionex 3000 was coupled to an TSQ Quantum™ Access MAX Triple Quadrupole Mass Spectrometer (Thermo Fisher, Waltham, MA, USA) equipped with an electrospray source. A Waters Acquity HSS T3 column 1.8 μm, 100 mm × 2.1 mm (Milford, MA, USA) was used for the separation of phenolics. Elution was at 40 °C, with a flow rate maintained at 0.4 mL/min. Eluent A consisted of 0.1% (*v*/*v*) formic acid in water, and eluent B consisted of 0.1% (*v*/*v*) formic acid in acetonitrile. Samples were eluted with the following gradient: 0.0–3.0 min from 2% to 20% B, 3.0–4.3 min at 20% B, 4.3–9 min from 20% to 45% B, 9–11 min from 45% to 100% B, 11–13 min at 100%, 13–15 min from 100% to 5% B. Calibration curves were constructed using chemical standards, and phenolics concentrations were expressed as mg/L of FL-BSG medium. Detection was performed in the multiple reaction monitoring (MRM) mode. Data acquisition was interfaced to a computer workstation running Xcalibur software version 4.1 (Thermo Fisher, Waltham, MA, USA). Chemical standards were used for phenolics identification by comparison of retention time and qualifier and quantifier ions. The results were expressed as means of three biological replicates analyzed in triplicate ± standard deviations.

### 2.7. RNA Isolation and Transcript Analysis by Quantitative Real-Time Polymerase Chain Reaction (RT-PCR)

Total RNAs were obtained from 1 mL of *L. plantarum* PU1 and *Leuc. pseudomesenteroides* DSM 20193 cells respectively grown in FL-BSG and MRS media at 30 °C until the LE phase of growth was reached after 14 and 8 h, respectively. Samples (ca. 8 log CFU/mL) were centrifuged (9000× *g* for 10 min at 4 °C) and RNA isolation was performed with Stool Total RNA purification kit as recommended by the manufacturer (Norgen, Thorold, ON, Canada) with some modifications. Cells were lysed using 200 μL of lysozyme 15 mg/mL and 20 μL of Proteinase K (Qiagen, Hilden, Germany) for 45 min at 25 °C under constant shaking (2000 rpm). Seven hundred μL of lysis buffer were added to the mixture and shaken vigorously. Five-hundred μL of propanol were used to precipitate the nucleic acids. Lysate was loaded into the column following the manufacturer’s instructions. Total RNA was treated with RNase-free TurboTM DNase (Ambion, Austin, TX, USA). Quality and quantity control of RNA was obtained on agarose-gel electrophoresis and by NanoDrop ND-1000 spectrophotometer (Thermo Fisher, Waltham, MA, USA), respectively.

Total RNA was transcribed to cDNA using random hexamers priming and the Tetro cDNA synthesis kit according to the manufacturer’s instructions (Bioline, London, UK). Primers for *L. plantarum* targeting *pbg6*, *pbg4* and *scrB* genes and for *Leuc. pseudomesenteroides* amplifying *INV*, *xylA* and *pbg*-like genes were used in this study. The specificity of the designed primer pairs was previously showed [22]. All reactions were set up in a QuantStudio 5 (Applied Biosystems, Germany) equipped with a 96 well reaction block. The reaction mixture (20 µL) contained 10 µL of TB Green™ Premix Ex Taq™ II (Tli RNaseH Plus) quantitative polymerase chain reaction (qPCR) master mix (Takara, Japan), 1 µL cDNA sample, and appropriate primer concentration (Table 1). Assays were carried out in triplicate on three biological replicates. PCR required an initial denaturation at 95 °C for 30 s, followed by a 40-cycle amplification consisting of denaturation at 95 °C for 5 s, annealing for 34 and 30 s for *Leuc. pseudomesenteroides* DSM 20193 and *L. plantarum* PU1, respectively, and the extension was for 34 and 40 sec, respectively (Table 1). Fluorescence signals were normalized according to ROX reference dye levels. After the last cycle of each amplification, a melt curve analysis, with a temperature range from 60 to 95 °C ramping at 1 °C/5 s, was performed to determine the product specificity. Gene expression data were normalized to levels of the 16S rRNA housekeeping gene and analyzed using a comparative cycle threshold method (ΔΔCT). Levels of expression of genes were compared using the relative quantification method [23,24]. Real-time data are shown as the relative change compared to *L. plantarum* PU1 and *Leuc. pseudomesenteroides* DSM 20193 grown in MRS. Error bars show the standard deviations (SD) of the ΔΔCT value. For relative quantification, the value of ΔCT for each sample was determined by calculating the difference between the value of CT of target genes and the value of CT of the 16S rRNA housekeeping gene. Then, the value of ΔΔCT for each sample was determined by subtracting the value of ΔCT of the calibrator (reference sample) from the ΔCT value for the sample. The normalized level of target gene expression was calculated by using the formula: 2 > ΔCT. A gene was considered overexpressed when its RE level was higher than 2 [25].

### 2.8. Statistical Analysis

Data were subjected to one-way or two-way analysis of variance (ANOVA), and pairwise comparison of treatment means was achieved by Tukey’s procedure at a *p* value of <0.05, using the Tukey–Krammer test through *HSD.test* function available in *agricolae* R package [26].

## 3. Results

### 3.1. Microbial and Biochemical Characterization of Raw FL-BSG

Total microbial cell density of FL-BSG sample was 2.97 ± 0.14 Log CFU/g. *Enterobacteriaceae* were 3.90 ± 0.14 CFU/g whereas *Enterococcus* and *Staphylococcus* were not found in 10 g of sample. Other spoilage microbial species, such as *Aeromonas, Pseudomonas* and coliforms, as well as acetic acid bacteria, yeasts and moulds were not found. Anaerobic bacteria were mostly facultative anaerobic in FL-BSG. Calcium, phosphor, iron and magnesium were found in FL-BSG at 69.4 ± 7.0, 40.8 ± 4.1, 52.6 ± 7.9 and 53.3 ± 5.4 mg/100 g, respectively. Potassium, manganese, and sodium concentration was lower (ca. 2–20%) compared to the other minerals assayed.

### 3.2. Kinetics of Growth and Organic Acids Production during FL-BSG Fermentation

**Figure 1:**
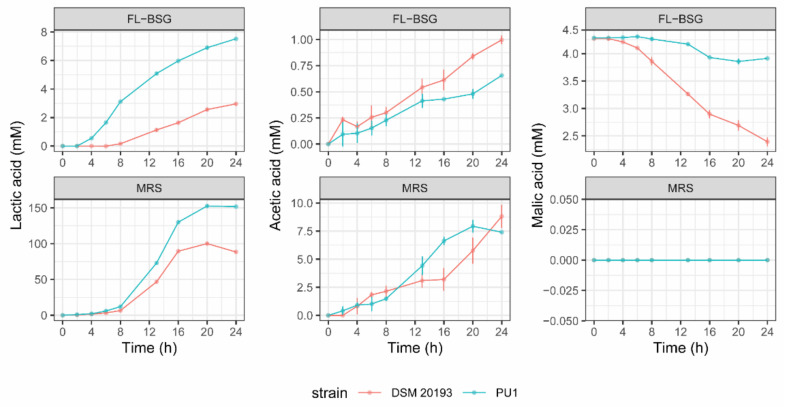
Organic acids production (mM) of *Leuconostoc pseudomesenteroides* DSM 20193 and *Lactiplantibacillus plantarum* PU1 during fermentation of Finnish brewers’ spent grain (FL-BSG) and MRS at 30 °C for 24 h.

Both strains grew under FL-BSG conditions showing a maximum cell density lower than 9.0 Log/CFU (Table 1). *Leuc. pseudomesenteroides* DSM 20193 had the highest maximum growth rate (µmax) in FL-BSG. A longer lag phase (λ) was found for *L. plantarum* PU1 compared to *Leuc. pseudomesenteroides* DSM 20193. As expected, when cultivated in the rich MRS medium, both bacteria showed an increase (ca. one and a half more log cycle) of the cell density compared to the cultivation in FL-BSG medium. The values of μmax and λ calculated based on the data modelling growth were almost consistent with the final cell densities. The greatest decrease of pH was reached by *L. plantarum* PU1 (1.32 ± 0.10) in FL-BSG medium, which had an initial pH of 6.65, followed by *Leuc. pseudomesenteroides* DSM 20193 (1.02 ± 0.06). Kinetics of organic acids production were determined throughout 24 h of growth in FL-BSG and MRS media (Figure 1), and the production rate was calculated as the slope of the linear regression for a time range. Lactic acid was always the major fermentation end-product. Both strains showed a single metabolic phase during FL-BSG fermentation.

*L. plantarum* PU1 produced lactic acid from 2 up to 20 h with a rate of 0.41 ± 0.01 mM/h with a final concentration (at 24 h) of 7.52 ± 0.18 mM. *Leuc. pseudomesenteroides* DSM 20193 began to produce lactic acid after 8 h of growth in FL-BSG up to 20 h (2.56 ± 0.07 mM) with a rate of 0.15 ± 0.01 mM/h, which corresponded to the beginning of the growth phase (0.55 ± 0.02 h^−1^). Bacterial strains began to produce acetic acid from 2 up to 24 h of FL-BSG fermentation, which reached a final concentration of 1.00 ± 0.03 and 0.66 ± 0.07 mM for DSM 20193 and PU1, respectively. The rate of acetic acid production was slightly higher for DSM 20193 (0.04 ± 0.00 mM/h) compared to PU1 (0.03 ± 0.00 mM/h). Malic acid had an initial concentration of 4.27 ± 0.02 mM in FL-BSG and was mainly consumed by *Leuc. pseudomesenteroides* DSM 20193 after 6 h of growth (0.09 ± 0.01 mM/h) with a final consumption of 1.88 ± 0.08 mM while PU1 showed a very low consumption with a rate of 0.02 ± 0.01 mM/h. When cultivated in MRS medium, the concentration of lactic and acetic acids markedly increased, but the ratio acetic—lactic acids was lower than FL-BSG. Due to the low levels of glucose, fructose and sucrose in FL-BSG media (Table S1), after 24 h of fermentation, these carbohydrates were below the limit of detection of the instrument.

### 3.3. Phenotypic Profiles Adaptation to Brewers’ Spent Grain Ecosystem

**Figure 2:**
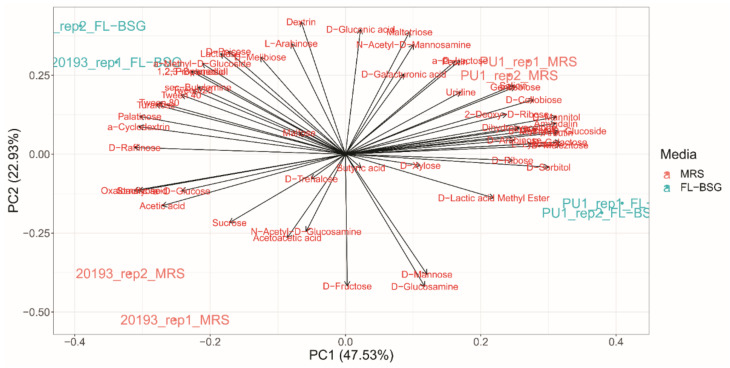
Principal component analysis (PCA) of the area under the curve (AUC) computed from phenotype microarray data for and *Lactiplantibacillus plantarum* PU1 and *Leuconostoc. pseudomesenteroides* DSM 20193 cultivated in Finnish brewers’ spent grain (FL-BSG) and MRS at 30 °C for 14 and 8 h. Loadings evidence substrate consumption across the PCA space.

**Figure 3:**
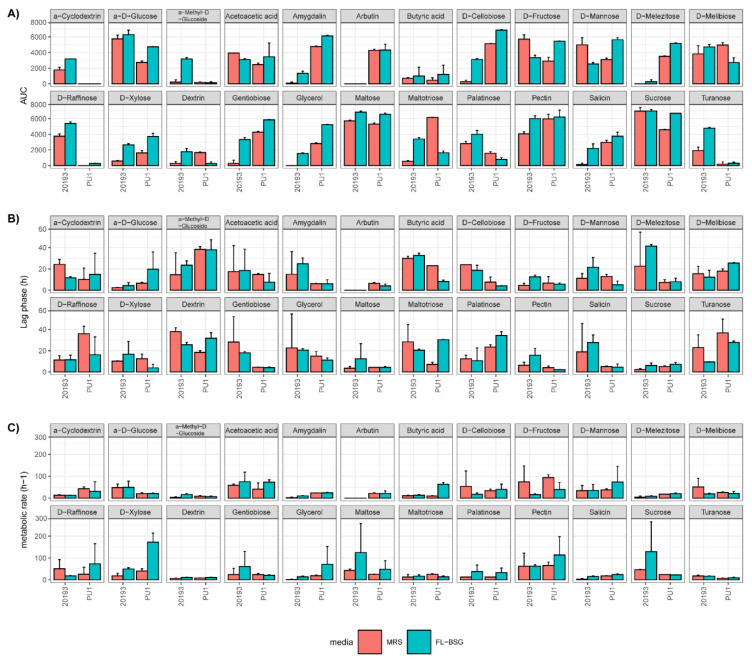
Metabolic parameters for selected substrates of *Lactiplantibacillus plantarum* PU1 and *Leuconostoc pseudomesenteroides* DSM 20193 (**A**) Area under the curve (AUC), (**B**) lag phase (h) and (**C**) metabolic tax (h^−1^) cultured in Finnish brewers’ spent grain (FL-BSG) and MRS during 14 and 8 h respectively, at 30 °C.

A phenotypic screen for *L. plantarum* PU1 and *Leuc. pseudomesenteroides* DSM 20193 was performed in FL-BSG medium and compared to the MRS through the Phenotype MicroArray OmniLog PM technology (Biolog) (Figure 2 and Figure 3). Cells were collected when the late exponential (LE) growth phase was reached and used to inoculate the PM plates. The range of phenotypes analysed included the transport, uptake, and catabolism of 190 carbon sources.

Principal component analysis of the area under the curve (AUC) computed from phenotype profile curves stratified the assayed conditions based on the genus and the culture medium (MRS and FL-BSG). Both strains showed a higher metabolic activity for a wider range of substrates when cultured in FL-BSG medium (global average AUC equal to 3024.8 ± 2215.3 OL·h) compared to MRS (average AUC equal to 2410.9 ± 2108.9 OL·h). The increase of activity was also reflected in higher metabolic rates and shorter lag phases for many compounds (Figure 3).

Starch and sucrose metabolism compounds suffered the highest fluctuations when strains were swap from MRS to FL-BSG. The cultivation of *Leuc. pseudomesenteroides* DSM 20193 in FL-BSG stimulated the consumption of oligosaccharides (i.e., α-cyclodexrin and pectin) when compared to PU1. Both strains increased their metabolism for D-cellobiose, which is linked to phospho-β-glucosidase activity found in these strains. Consequently, other β-glucosides such as gentiobiose, salicin and amygdalin were highly degraded under FL-BSG conditions. PU1 was the only strain able to degrade arbutin. The growth on FL-BSG medium stimulated the metabolism of maltose and maltotriose for both strains. Sucrose metabolism was stimulated when PU1 was cultured in BSG while DSM 20193 did not show a significant change when compared to MRS. However, the metabolism drift under FL-BSG of sucrose isomers was specie-dependent. The metabolism of D-threalose, turanose and D-melezitose were highly degraded by both strains, where DSM 20193 preferred turanose and the overall metabolism of melezitose was more intense in the PU1 strain. Contrastingly, the cultivation of DSM 20193 in BSG medium stimulated palatinose degradation but not for PU1, whose metabolism was found to be higher in MRS.

Fructose and mannose metabolism was more intense for *L. plantarum* PU1 when cultured on FL-BSG. *Leuc. pseudomesenteroides* DSM 20193 preferred mannitol while no significant (*p* < 0.05) differences were found for PU1 (Figure 3). Raffinose and its degradation product, melibiose, were also found to be highly consumed by *Leuc. pseudomesenteroides* DSM 20193 but no significant activity was found by *L. plantarum* PU1. Substrates involved in pentose and glucoronate interconversions pathway (e.g., xylose, D-galacturonic acid and pectin) were also highly metabolized in the BSG compared to the MRS condition by both strains. Glycerol was also degraded by both strains under FL-BSG.

### 3.4. Phenolic Profile Changes during BSG Fermentation

**Figure 4:**
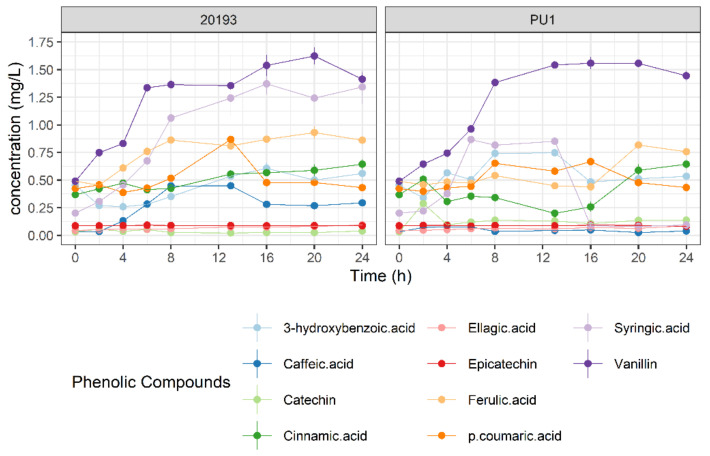
Profile evolution of phenolic compounds of Finnish brewers’ spent grain (FL-BSG) fermented with *Lactiplantibacillus plantarum* PU1 and *Leuconostoc pseudomesenteroides* DSM 20193 for 24 h at 30°.

We further investigated how the FL-BSG phenolic profile changed during 24 h of fermentation (Figure 4). Among 10 identified phenolic compounds, vanillin and syringic acid increased during the FL-BSG fermentation. The increase of vanillin occurred linearly during the first 6–8 h with a rate of 0.15 ± 0.01 and 0.13 ± 0.01 g/L·h for DSM 2013 and PU1. Analogously, DSM 2013 released syringic acid during the first 8 h with a rate of 0.10 ± 0.01 g/L·h while no consistent release of syringic acid was observed for *L. plantarum* PU1. A mild release of ferulic acid was found for DSM 20193 when cultured in FL-BSG after 4 h of incubation (0.05 ± 0.00 g/L·h) while the release of ferulic acid by *L. plantarum* PU1 occurred after 16 h of fermentation with a rate of 0.09 ± 0.00 g/L·h.

### 3.5. Gene Expression Coding for Galactose, Sucrose and Starch and Glucuronate Interconversions Pathways under Brewers’ Spent Grain Conditions

Quantification of the expression of 6 genes involved in sucrose and its isomers metabolism (sucrose-6-phosphate hydrolases), and pentose and glucoronate interconversions pathway (xylose isomerase) was aimed to determine whether particular pathways are over-expressed in response to the BSG substrate (Table 2). Phospho-β-glucosidase activities were also targeted for putative enzymes involved in the degradation of cellobiose (*pbg4* and *pbg6* related genes) and other β-glucosides, such as gentiobiose and glycosylated forms of polyphenolic compounds (*pbg8* gene).

*L. plantarum* PU1 over expressed *ScrB* (64.5 ± 14.6-fold), *pbg4* (27.2 ± 2.2-fold) and *pbg6* (70.8 ± 9.6-fold) genes during growth in FL-BSG conditions. By contrast, no overexpression was found for *pbg8* gene. *Leuc. pseudomesenteroides* DSM 20193 showed an overexpression of *xylA* (9.36 ± 3.36-fold) and *INV* (19.02 ± 3.9-fold) genes after 14 h of FL-BSG fermentation. *Pbg4-like* gene, which encodes for a 6-phospho-beta-glucosidase was also differentially expressed (7.03 ± 2.68-fold) under FL-BSG conditions.

## 4. Discussion

In the present study we provided insights into the metabolism of carbon sources and, particularly, of β-glycosides in *L. plantarum* and *Leuc. pseudomesenteroides* during brewers’ spent grain (BSG) fermentation. Currently, BSG exploitation as a food ingredient is still limited due to its poor technological and sensory performance. High lignocellulosic content causes astringent flavors and woody texture properties. Effective reduction/modification of cellulose-derived and phenolic compounds in BSG might be achieved through an accurate selection of LAB starters with targeted metabolic activities [2,11]. *L. plantarum* and *Leuc. pseudomesenteroides* were used as model heterofermentative LAB because of their presence in plant niches and their role in bioprocessing of plant-based foods [2,27]. The production of dextrans by *Leuc. pseudomesenteroides* DSM 20193 and its positive impact upon plant-matrices technological and sensory properties was a promising metabolic trait [28,29,30]. The largest genome sizes (1.93 Mb) of *Leuc. pseudomesenteroides* among 17 subspecies of *Leuconostoc* genus suggests a wide panel of metabolic routes and fitness to plant ecosystems [31]. On the other hand, the paradigm of nomadic lifestyle is represented by *L. plantarum* since it did not undergo reductive evolution [32,33]. One example of its metabolic flexibility is the high redundant presence of phospho-β-glycosidase encoding genes [34,35]. Furthermore, the capability of *L. plantarum* PU1 to hydrolyze arabinoxylans present in aleurone BSG cell walls and to release peptides with high radical scavenging activity was previously demonstrated, suggesting its ability to improve the BSG technological and sensory features [12]. To the best of our knowledge, first this study showed a whole metabolic profiling, emphasizing phospho-β-glycosidase activities, from *L. plantarum* and *Leuc. pseudomesenteroides* under the BSG ecosystem compared to a standard laboratory medium. Despite the important role of LAB to exploit cereal by-products, few studies characterize in-depth LAB metabolism during lignocellulosic-like substrates. Our approach combined a whole-phenome characterization together with a set of metabolome analyses and differential gene expression to obtain insights into the metabolism of LAB related to the main constituents of BSG. The main minerals found were in agreement with previous studies, being calcium, magnesium and phosphorous the most abundant, where the total mineral content was slightly higher [36]. The growth of facultative bacteria and *Enterococcus* spp. on BSG might indicate a post-production proliferation [37].

Phenotype profiling unveiled the differentiation of metabolism of the strains under BSG and MRS standard condition, which reflected the effect of these contrasting environmental conditions. Metabolic performance was enhanced for a wider number of carbon sources under BSG conditions, evidencing the heterologous variety of complex carbohydrates present in BSG [11,37]. This is also casted back in the higher ratio of acetic/lactic acid found for both strains under BSG compared to MRS and the consumption of malic acid to counteract stressful conditions.

Celluloses are the main constituent of BSG husk [11]. Although lactic acid bacteria have a limited cellulosic machinery, the *L. plantarum* WCFS1 genome harbors a putative endoglucanase (*lp_3433*) and eleven genes encoding for phospho-β-glycosidases activities [35,38]. Recently, we showed that phospho-β-glycosidases encoded in *L. plantarum* and *Leuc. pseudomesenteroides* DSM 20193 genomes diverged according to the specificity of the PTS complex they were associated, remarking their specificity for panel of β-glycosides substrates [22]. Phenotype profiling revealed an enhanced metabolism of gentiobiose and cellobiose under BSG conditions, which was reflected in shorter lag phases and higher metabolic rates. These preliminary findings were confirmed by gene expression analyses. *L. plantarum* PU1 significantly increased the expression of *pbg6* and *pbg4* genes while *Leuc. pseudomesenteroides* DSM 20193 showed a higher fold change for a *pbg*-like gene. These genes are allocated in their respective operons along with cellobiose-specific PTS. The PTS systems associated to 6-phospho-β-glucosidases encoding genes showed in *L. acidophilus* a clear segregation depending on the induction by cellobiose or gentiobiose [39]. Notably, cellobiose and gentiobiose are composed of two glucose units only differing in the glycosidic linkage, β-(1→4) or β-(1→6) respectively. Our results highlight the importance of cellobiose metabolism linked to cellulosic substrates like BSG. We hypothesized that cellobiose is a pivotal carbon source for the growth and surveillance of *L. plantarum* and *Leuc. pseudomesenteroides* during BSG fermentation. Nonetheless, further investigation on the specificity of LAB β-glucosides PTS systems and 6-phospho-β-glucosidases is needed to establish their specificity towards other β-linked disaccharides such as gentiobiose [40,41].

Because β-glycosidated phenols have a low bioactivity, LAB 6-phospho-β-glucosidases have demonstrated increasing nutritional properties of plant-based foods through the release of their respective aglycones [5,42]. Phenotyping revealed an increased consumption of salicin and amygdalin for both bacterial strains, and arbutin in the case of *L. plantarum* PU1, although no significant fold expression was found for the generic β-glycoside-associated *pbg8* gene for PU1 strain. Transcriptomic of *L. plantarum* cultured in fruit juices reported that *pbg8* was overexpressed after 30 days of maintenance and not during the growth phase [43]. Notably, a release of syringic acid and vanillin occurred during BSG fermentation with both bacterial strains. Most of the phenolic compounds found in BSG are related to lignin macromolecules, which are mainly constituted of vanillyl, syringyl and guaiacyl alcohols [44]. In plants, the monolignols synthesis is performed through the phenylpropanoid pathway where β-glycosilated conjugates are stored in the vacuole [45,46]. Hence, we hypothesized that β-glucosylated monolignols are degraded through 6-phospho-β-glucosidases during BSG fermentation and the resulting syringic acid is the result of specific benzyl alcohol dehydrogenase and aldehyde dehydrogenases [47,48]. *Leuc. pseudomesenteroides* DSM 20193 harbours only a phospho-β-glucosidases cellobiose-associated PTS system within the same operon suggesting a wider specificity of this transporter. In fact, previous studies demonstrated that generic β-glucoside PTS are essential for *L. plantarum* growth on fructooligosaccharides suggesting a wider specificity beyond β-glucosides [49,50]. Notably, degradation of β-fructosides caused an upregulation of *pbg10* gene [51].

The presence of sucrose in BSG may results from residual endosperm starch [11,37]. We found an increase of metabolism for maltose, sucrose and/or its plant-derived isomer trehalose under BSG conditions. These findings correlated with the observed increase of fold-change for sucrose-6-phosphate hydrolase for both bacterial strains and the raw content of sucrose in the BSG. Furthermore, this enzymatic activity might also be involved in the degradation of anti-nutritional factors, such as raffinose, naturally present in BSG. This would explain the metabolic activity observed for this substrate by DSM 20193 under BSG conditions [37].

Arabinoxylans are the most abundant pentosans in BSG [37]. Phenotyping unveiled a high metabolic activity of xylose for both strains. In the case of *Leuc. pseudomesenteroides* DSM 20193, this finding agrees with the marked fold change of *xylA* gene. This strain harbors a *xynB* gene encoding for a 1,4-β-xylosidase, which enables it to cleave xylo-oligosaccharides [52]. Conversely, *L. plantarum* does not apparently encode for any gene for the degradation of xylosides. However, previous reports suggest a widespread presence of *csc* gene clusters encoding for cell surface protein complexes with ConA-like domains, which would be involved in binding and/or degradation of complex (plant-derived) oligo- or poly-saccharides [53].

In this study, we provided a comprehensive view of the important role exerted by 6-phospho-β-glucosidases of *Leuc. pesudomesenteroides* DSM 20193 and *L. plantarum* PU1 as well the major metabolic routes undertaken during plant-based fermentation. Further challenges will consider a controlled characterization of *pbg* expression correlated to the metabolism of β-glucosides with different aglycone moieties.

## Figures and Tables

**Table 1:** Parameters of the growth kinetic of *Lactiplantibacillus plantarum* PU1 and *Leuconostoc pseudomesenteroides* DSM 20193 strains during fermentation of Finnish brewers’ spent grain (FL-BSG) and Rich De Man, Rogosa and Sharpe (MRS) media at 30 °C for 24 h.

Media ^a^	Species/Strain	Growth ^b^		
		A	μmax	λ
FL-BSG	*Lactiplantibacillus plantarum* PU1	8.19 ± 0.03 ^C^	0.27 ± 0.02 ^C^	4.03 ± 0.17 ^C^
	*Leuconostoc pseudomesenteroides* DSM 20193	8.10 ± 0.06 ^D^	0.40 ± 0.03 ^C^	3.30 ± 0.08 ^B^
MRS	*L. plantarum* PU1	9.73 ± 0.03 ^A^	0.53 ± 0.01 ^B^	2.38 ± 0.06 ^A^
	*Leuc. pseudomesenteroides* DSM 20193	9.53 ± 0.03 ^B^	0.62 ± 0.01 ^A^	1.47 ± 0.06 ^C^

^a^ For the manufacture of the media, see Materials and Methods. ^b^ Growth data were modelled according modelled according to the logistic equation available in grofit R package [16]. Parameters for growth: A, maximum absorbance reached by the culture at the stationary phase of growth (log CFU/mL); µmax, maximum growth rate (log CFU/mL·h); λ, length of the lag phase (h). Means within the columns followed by different letters (A to D) are significantly different (*p* < 0.05). Shown are mean values ± standard deviations for the three batches of each type of media, analyzed in duplicate.

**Table 2:** Relative expression of selected genes of *Leuconostoc pseudomesenteroides* DSM 20193 and *Lactiplantibacillus plantarum* PU1 cultivated on Finnish brewers’ spent grain (FL-BSG) until the late (after ca. 14 h) exponential (LE) phase of growth at 30 °C was reached. The calibrator conditions used were the same bacterial cultures grown in MRS until the LE phase of growth (after ca. 8 h) at 30 °C was reached.

Strain	Gene	Culture media	Fold Change
*Lactiplantibacillus plantarum* PU1	*pbg4*	FL-BSG	27.18 ± 2.17
		MRS	1.00 ± 0.09
	*pbg6*	FL-BSG	70.76 ± 9.62
		MRS	1.01 ± 0.15
	*scrB*	FL-BSG	64.47 ± 14.6
		MRS	1.41 ± 1.01
*Leuconostoc pseudomesenteroides* DSM 20193	*pbg*-like	FL-BSG	7.03 ± 2.68
		MRS	1.00 ± 0.12
	*INV*	FL-BSG	9.36 ± 3.36
		MRS	1.01 ± 0.18
	*xylA*	FL-BSG	19.02 ± 3.9
		MRS	1.00 ± 0.03

Data are the means of three biological replicates analyzed in triplicate ± standard deviations.

## Data Availability

Not applicable.

## Supplementary Materials

The following are available online at 10.5281/zenodo.4295968, Table S1: Main chemical composition of the culture media. The data are the means of three independent experiments ± standard deviation, analysed in duplicate. n.d., not detected.
